# Diverse floral scents in Malagasy *Bulbophyllum* evolve in a bounded fashion

**DOI:** 10.3389/fpls.2026.1785538

**Published:** 2026-03-24

**Authors:** Silvia Artuso, Alexander Gamisch, Anton Sieder, Roman Fuchs, Sven Gindorf, Mario Schubert, Stefan Dötterl, Hans Peter Comes

**Affiliations:** 1 Department of Environment and Biodiversity, University of Salzburg, Salzburg, Austria; 2 Core Facility, Botanical Garden, University of Vienna, Vienna, Austria; 3 Department of Biology, Chemistry and Pharmacy, Freie Universität Berlin, Berlin, Germany

**Keywords:** *Bulbophyllum*, chemical ecology, floral scent, Orchidaceae, phylogenetic comparative methods, plant–pollinator interactions, selective constraints

## Abstract

Floral scent is a fundamental feature in angiosperm pollination, reproductive isolation, and speciation. However, little is known about the neutral or selective processes shaping long-term patterns of floral scent evolution within particular lineages. Here we investigated the floral scent composition and variation of 41 species of Malagasy *Bulbophyllum* orchids (crown age: *c*. 12.7 Ma). In addition, we inferred the mode of floral scent evolution across 32 species of this lineage, using phylogenetic comparative methods (PCMs) that represent a novel approach to this field of research. Both qualitative and semi-quantitative variability of floral scent was high among species. Of the 297 volatile organic compounds (VOCs) detected, 225 (*c*. 76%) were species-specific, including a novel floral scent compound (N,N-dimethylleucine O-methyl ester), and the relative scent composition varied significantly among subclades. Whole scent bouquets but also most single VOCs best fitted a constrained (Ornstein-Uhlenbeck) model of trait evolution. The high diversity and specificity of floral scent compounds compounds observed, with some known to be bioactive during orchid pollination, suggests that scent has an important role in the pollinator attraction of Malagasy *Bulbophyllum*. Our results further support the hypothesis that floral scent has not evolved neutrally in this lineage but in a bounded fashion, possibly due to strong selective constraints imposed by the group’s main pollinators (small Dipteran flies).

## Introduction

Floral scents, produced by flowering plants (angiosperms), are a highly diverse group of volatile organic compounds (VOCs) that play a crucial role in attracting animal pollinators (Dötterl and Gershenzon, 2023). The most studied angiosperm family in terms of floral scent is Orchidaceae, with about a thousand different VOCs described (Knudsen et al., 2006; Perkins et al., 2023). Several of these have been found or suggested to play a role in orchid pollination, reflecting the high diversity of chemically mediated interactions between orchids and their pollinators (reviewed in Perkins et al., 2023; see also Liu et al., 2025), while they might additionally or alternatively be also involved in other functions, such as deterring antagonists or fighting pathogens (Dötterl and Gershenzon, 2023). Both rewarding and deceptive orchids utilize VOCs to attract pollinators (e.g. Huber et al., 2005; Salzmann et al., 2007; Schiestl and Schlüter, 2009; Vereecken et al., 2010; Slavković and Bendahmane, 2025). For example, some deceptive orchids mimic the sex pheromones of female insects to dupe male insects (Peakall, 2023), while others mimic egg-laying sites of their pollinators (Jürgens et al., 2013). In some rewarding orchids, floral scents not only act as attractants for pollinators but also as rewards, for example, in species pollinated by male euglossine bees collecting floral fragrances for mating purposes (Henske et al., 2023). In some orchids, floral scent has been shown to be under pollinator-mediated selection (Stökl et al., 2008; Gross et al., 2016; Chapurlat et al., 2019), and changes in scent composition have also been suggested to play a key role in pollinator-driven speciation by mediating pre-zygotic reproductive isolation (RI) (e.g. Gervasi and Schiestl, 2017; Ackerman et al., 2023). However, to date, most studies on floral scent in orchids have focused mainly on the functional, pollination-ecological and/or microevolutionary (e.g. RI) roles of different VOCs in one or a few target species (Perkins et al., 2023; Schwery et al., 2023). By contrast, phylogenetic comparative studies on floral scent variation are rare in orchids, as is generally true for angiosperms (but see Ramírez et al., 2011; Kite and Hetterscheid, 2017; Joffard et al., 2020; Liu et al., 2024). In consequence, there is not only a general lack of knowledge about floral scent variation within specific orchid or other angiosperm lineages but also a limited understanding of the ultimate evolutionary forces (neutral vs selective) driving the diversification viz. ‘disparification’ of entire scent bouquets, or components thereof, over macroevolutionary (i.e. million-year) time scales (Perkins et al., 2023; Schwery et al., 2023). Clarifying these issues is also crucial for gaining an in-depth understanding of the tempo and mode of scent evolution and the role of VOCs, either alone or together with other floral (e.g. visual) signals, in mediating plant–pollinator interactions and, potentially, species diversification (e.g. Ackerman et al., 2023).

Members of the pantropical and mostly epiphytic orchid genus *Bulbophyllum* Thouars (Epidendroideae; *c.* 2,200–2,400 spp.; POWO, 2025) provide particularly interesting model systems to address these issues. Most species of this mega-diverse genus are adapted to cross-pollination by flies or rarely bees (e.g. Pridgeon et al., 2014; Gamisch et al., 2016; Wiśniewska et al., 2023). Their floral scent is usually produced by osmophores on perianth organs, including the labellum or ‘lip’ (e.g. Teixeira et al., 2004; Davies and Stpiczyńska, 2014; but see Davies et al., 2025) and is used to attract the insects to the flower and guide them towards the gynostemium/column (i.e. the fused male/female compound structure of orchids) for pollinia removal or deposition (Tan et al., 2006; Nakahira et al., 2018; Tan and Tan, 2018; Wiśniewska et al., 2023). Previous studies point at considerable interspecific floral scent variation in *Bulbophyllum* (e.g. Kaiser, 1993; Chen et al., 2005; Humeau et al., 2011; Wiśniewska et al., 2023), including differences between fly- vs bee-pollinated species from the Neotropics (Silva et al., 1999) or among taxa from Southeast Asia (e.g. *B. baileyi*, *B. cheiri*, *B. macranthum*, *B. patens, B. sinapis*; Tan and Nishida, 2000, Tan and Nishida, 2005; Nakahira et al., 2018). In these latter *Bulbophyllum* species, the main compounds (e.g. methyl eugenol, raspberry ketone, zingerone) they produce act as both attractants and rewards for fruit-fly males (e.g. *Bactrocera*, Tephritidae/Dacini), which in turn use these compounds to boost their defences and as precursors to produce sex pheromones (see also Katte et al., 2020; Tan et al., 2023). Most recently, Davies et al. (2025) reported a total of 44 VOCs, mainly fatty acid derivatives, across five *Bulbophyllum* species of the mainly Asian ‘*Cirrhopetalum* alliance’, with the number of compounds ranging between five and 15 per species.

In the present study, we investigated patterns of floral scent composition, variation and evolution among species of *Bulbophyllum* native to the humid to seasonally dry rainforests of Madagascar. Based on phylogenetic-biogeographical evidence (Gamisch and Comes, 2019; Gamisch et al., 2021), *Bulbophyllum* colonized this tropical island only once, *c*. 12.7 million years ago (Ma), resulting in a spectacular radiation of *c*. 210 species/16 sections (Sieder et al., 2009; Madagascar Catalogue, 2025), with some species also occurring in the Comoros, the Mascarenes (La Réunion, Mauritius) or the African mainland (e.g. Fischer et al., 2007; Hermans et al., 2021). Studies on trait evolution in this monophyletic ‘Malagasy *Bulbophyllum* lineage’ (*sensu* Gamisch and Comes, 2019) have so far focused on aspects of breeding system (outcrossing/selfing; e.g. Gamisch et al., 2015), photosynthetic pathways (C_3_, CAM) in relation to forest habitat preference (Gamisch et al., 2021), as well as three-dimensional (3D) flower shape variation and modularity (Artuso et al., 2021, Artuso et al., 2022). As typical for the genus, species from Madagascar possess a specialized fly-pollination mechanism based on a hinged and mobile labellum that acts as a trap for the insect, forcing it into contact with the column for pollinia removal or deposition (Artuso et al., 2022, and references therein). However, field observations on pollinators are currently limited to small Dipteran flies visiting flowers of *B. cardiobulbum* in Madagascar (Chloropidae/*Arcuator* spp.: Hermans et al., 2021) and *B. variegatum* and *B. mascarenense* in La Réunion (e.g. Platystomatidae sp.: Humeau et al., 2011; Mycetophilidae sp.: Pailler and Baider, 2020). Likewise, there are very few studies on floral volatiles in *Bulbophyllum* from Madagascar and adjacent islands. For example, Humeau et al. (2011) reported a sapromyiophilous pollination system with urine-like unpleasant odour among accessions of *B. variegatum* (sect. *Alcistachys*) from La Réunion, which could be attributed to indole, *p*-cresol and 2-heptanone. In addition, preliminary surveys among five closely related *Bulbophyllum* species of sect. *Calamaria* from Madagascar revealed considerable variation in dominant scent compounds (i.e. *B. bicoloratum:* undecane; *B. obtusatum*: ethyl dodecanoate; *B. occultum*: 1-octanol, isogeraniol; *B. senghasii*: methyl salicylate isomer; *B. trifarium*: tridecane; T. Grasegger & S. Dötterl, unpubl. data). Again, most of these compounds suggest that flies associated with carrion or excrement are the most likely pollinators (Dobson, 2006). It is feasible, therefore, that floral scent evolves rapidly (and perhaps largely unconstrained) in Malagasy *Bulbophyllum* as potential prezygotic RI mechanism driving pollinator-mediated speciation (e.g. Schiestl and Schlüter, 2009; Ackerman et al., 2023; Schwery et al., 2023). However, testing this hypothesis requires the analysis of scent variation across a wider taxon sampling by also taking phylogenetic relationships into account.

Accordingly, the major aim of the present work is to provide further insights into the composition and variation of floral scent in Malagasy *Bulbophyllum*, based on a survey of 41 species (50 accessions), representing nine (of the 16) sections. In addition, we employed phylogenetic comparative methods (PCMs; Garamszegi, 2014), including multivariate tests of phylogenetic signal (Adams, 2014) and models of continuous trait evolution (Morlon et al., 2016; Clavel et al., 2019), to study floral scent evolution across a subset of 32 species with known genealogical relationships, based on the time-calibrated nuclear/plastid phylogeny of Malagasy *Bulbophyllum* (Gamisch et al., 2021). Hence, to our knowledge, this study is the first to employ PCM-based modelling to investigate whether floral scent in an angiosperm lineage evolved according to a neutral Brownian ‘random walk’ (i.e. at a constant rate and without directionality) or in a selectively constrained (‘bounded’) fashion.

## Materials and methods

### Taxon sampling and phylogenetic background

Our floral scent dataset of Malagasy *Bulbophyllum* includes a total of 41 species (50 accessions), representing 30 recognized species plus 11 potentially new ones (*B.* sp.; **Supplementary Table 1**). Based on previous phylogenetic data (Gamisch et al., 2021) and/or morphological evidence, these 41 species represent nine of the 16 sections and all main clades of this island radiation, i.e. clade *A* (12 out of 50 spp.; sects. *Calamaria* and *Kainochilus*; crown age: *c*. 11.6 Ma), clade *B* (28/126 spp.; sects. *Elasmotopus*, *Lichenophylax*, *Loxosepalum*, *Pachychlamys*, *Ploiarium*, and *Trichopus*; *c*. 10.9 Ma), and clade *D* (1/3 spp.; only sect. *Inversiflora*; *c.* 12.7 Ma). Since most of the sampled clade *B* species belong to the non-monophyletic sect. *Ploiarium* (*n* = 19), they were treated separately from the rest of this clade in some of the statistical analyses (see below).

### Plant material, volatile collection and compound identification

We sampled floral scents emitted from flowering individuals cultivated at the University Botanical Gardens of Vienna (HBV; *n* = 38) and Salzburg (HBS; *n* = 3), respectively, or growing *in situ* in Madagascar (*n* = 9; January/February 2018; see **Supplementary Table 1** for voucher numbers as well as date, duration, and locality of scent sample collections). Although we did not test for effects of growing condition (*in situ* vs *ex situ*) and site on the floral scent emission of the studied *Bulbophyllum* species, data on other orchids and non-orchids suggest that these factors have very limited effects on scent compositions (Heiduk et al., 2016; Messado et al., 2025). Floral volatiles were collected using dynamic headspace following Dötterl et al. (2005). In brief, a single inflorescence per individual was carefully enclosed with a polyethylene oven bag (Toppits ^®^, Melitta, Germany), and a glass tube (25 mm long, 2.2 mm outer diameter; Hilgenberg GmbH, Maisfeld, Germany), containing silanized glass wool and the adsorbents Carbotrap B (mesh 20/40) and Tenax TA (60/80) (1.5 mg each; both Supelco/Sigma-Aldrich, Bellefonte, PA, USA), was inserted in the bag. Air enriched with floral scent was extracted through the tube using an electric pump (Gardner Denver Thomas GmbH, Memmingen, Germany) at 200 ml/min. The floral headspace was preferably captured in the morning, i.e. when day-flowering and fly-pollinated *Bulbophyllum* species usually receive most insect visits in the wild (e.g. Tan et al., 2002; Hu et al., 2017; Nishida et al., 2022). For glasshouse plants, scent collection was initially performed for 20–30 min, depending on the numbers of flowers and the intensity of odour. However, after observing a low amount of scent in most of our study species, we increased the sampling time to 150 minutes. In the field, sampling was carried out for 5–30 min (see **Supplementary Table 1**). For each accession, volatiles from ambient air and leaves were collected as negative controls.

All volatile samples were stored in a fridge at -20 °C until they were analysed using thermal desorption gas chromatography/mass spectrometry (TD-GC/MS; Marotz-Clausen et al., 2018). Obtained data were analyzed using GCMSolution v.4.4.1 (Shimadzu Corporation, Kyoto, Japan). Compounds were identified based on linear retention indices using a series of *n*-alkanes (C_7_–C_20_; Van den Dool and Kratz, 1963) and by comparing their mass spectra with data available in the databases ADAMS, ESSENTIALOILS-23P, FFNSC 2, and W9N11 (see also Rupp et al., 2024). If possible, compound identities were confirmed by using retention indices and mass spectra of authentic standards available at the Plant Ecology Lab of Salzburg University. A previously unknown floral scent compound, which dominated the scent of *B. cardiobulbum* (clade *D*), was further analysed by Nuclear Magnetic Resonance (NMR) spectroscopy and identified as N,N-dimethylleucine O-methyl ester (see **Supplementary Methods S1** for details and chemical structure). The absolute scent amounts (total ion current) from accessions belonging to the same species were then averaged to obtain one scent profile per species for all subsequent analyses.

### Statistical comparison of scent profiles between clades/sections

Based on the VOC dataset of the 41 species, we tested whether the number of compounds differed significantly between members of clade *A* and *B* and between clade *A*, sect. *Ploiarium* of clade *B*, and the ‘rest of clade *B’*. A Shapiro–Wilk normality test in R v.4.4.0 (R Core Team, 2024), coupled with visual inspection (QQ plots and histograms), revealed non-normality in the data. Consequently, we performed the above group comparisons using non-parametric Wilcoxon and Kruskal–Wallis rank sum tests as implemented in the stats R package (R Core Team, 2024). For our comparative multivariate (ordination) analyses of floral scent variation, we first calculated for each species the relative abundance of each compound (i.e. the peak area of each VOC divided by the peak area of all VOCs in the sample) using vegan v.2.6-6 (Oksanen et al., 2019; function *decostand*). Based on this dataset, we generated a Bray–Curtis dissimilarity matrix (vegan function *vegdist*, method = ‘bray’) to perform a nonmetric multidimensional scaling (NMDS) analysis for illustrating similarities and differences in scent composition among the 41 species (vegan function *metaMDS*). Results were plotted in two dimensions using ggplot2 v.3.5.1 (Wickham, 2016). Floral VOCs significantly associated with the first two NMDS axes (*P* < 0.05) were identified using the vegan function *envfit*. Permutational multivariate analysis of variance (PERMANOVA; vegan function *adonis*) was used to assess the significance of the factor ‘clade/section’ (i.e. clade *A*, sect. *Ploiarium* of clade *B*, and rest of clade *B*) on the scent composition. In addition, we conducted a permutational analysis of multivariate dispersions (PERMDISP) to test whether the three groups differed significantly in the dispersion (variability) of their scent profiles (vegan function *betadisper*) based on their spatial medians. Pairwise group comparisons for PERMANOVA were performed using pairwiseAdonis v.0.4.1 (Martinez Arbizu, 2020) with Bonferroni-adjusted *P*-values and for PERMDISP using the vegan function *permutest*. All the above significance tests used 999 permutations.

### Testing for phylogenetic signal and models of scent evolution

For our macroevolutionary (PCM) analyses of scent variation, we first pruned the full 179-spp. maximum clade credibility (MCC) chronogram of Malagasy *Bulbophyllum* (Gamisch et al., 2021) in phytools v.2.1-1 (Revell, 2012; function *drop.tips*) to obtain a phylogeny of 32 species for which these data were available. These species (highlighted bold in **Supplementary Table 1**) represent nine (of the 16) sections of this lineage and its three major clades (i.e. *A*, *B* and *D*; see also **Supplementary Figure S1**). We first tested for phylogenetic signal on the relative abundances of VOCs shared by at least two species (59 VOCs) using a multivariate version of Blomberg’s *K*-statistic (*K*_mult_; Adams, 2014), as implemented in geomorph v.4.0.7 (Adams et al., 2020; function *physignal*). Values of *K*_mult_ range from 0 to infinity and can be used to test if species tend to resemble one another more (0 < *K*_mult_< 1) or less (*K*_mult_ > 1) than expected under a Brownian motion (BM) model of trait evolution (*K*_mult_~ 1; Blomberg et al., 2003; Ackerly, 2009; see also Heiduk et al., 2017; Artuso et al., 2021; Liu et al., 2024).

In addition, we used the function *fit_t_pl* in rpanda v.2.3 (Morlon et al., 2016), based on a penalized likelihood (PL) framework (Clavel et al., 2019), to fit three commonly used PCM-based models of trait evolution, including BM, ‘early burst’ (EB) and ‘single-optimum Ornstein-Uhlenbeck’ (OU) models, to the matrix of relative abundances of the VOCs shared by at least two species to assess the mode of scent evolution across the pruned 32-spp. phylogeny. Note that the EB model describes an initially rapid phenotypic evolution followed by a slowdown (Harmon et al., 2010). By contrast, the OU model constrains the constant-rate ‘random walk’ (or neutral) BM process by including a strength of selection parameter (*α*) viz. an evolutionary force (‘pull’) toward an optimal trait value (e.g. Hansen, 1997; Beaulieu et al., 2012; Cressler et al., 2015). However, if the value of *α* is small (< 2; Beaulieu et al., 2012) and/or its associated ‘phylogenetic half-life’ (*t*_1/2_ = ln2/α; Hansen, 1997, Hansen, 2014) is larger than the total timespan (*t*) of the phylogeny, the OU model is no longer distinguishable from the BM model (Cooper et al., 2016; see also Artuso et al., 2022). We estimated *t*_1/2_ by assuming *t* = *c*. 12.7 million years (Myr), based on the crown age of the 32-spp. phylogeny (see **Supplementary Figure S1**). For inferring the best-fit model (BM, EB or OU), we used the lowest score of the Generalized Information Criterion (GIC; rpanda function *GIC*).

Finally, we fitted the same macroevolutionary models (BM, EB and OU) to the relative abundances of each of the VOCs separately in geiger v.2.0.11 (Pennell et al., 2014; function *fitContinuous*) to test whether they had evolved according to the same or different models. Initial analyses using default parameter ranges resulted in estimated parameters reaching their bounds for the OU and EB models (data not shown). Hence, following Pennell et al. (2014), search intervals were adjusted manually where needed (i.e. upper *α* = 10.0 for OU, lower *r* = -1.0 and upper *r* = 1.0 for EB). The model with the lowest score of the size-corrected Akaike Information Criterion (AICc) was considered the ‘best’, whereas those in which the difference to the best-fit model (ΔAICc) was < 2 were regarded to be equivalent (Burnham and Anderson, 2002; see also Gamisch et al., 2021).

## Results

### General patterns of floral scent variation and compound identification

**Figure 1:**
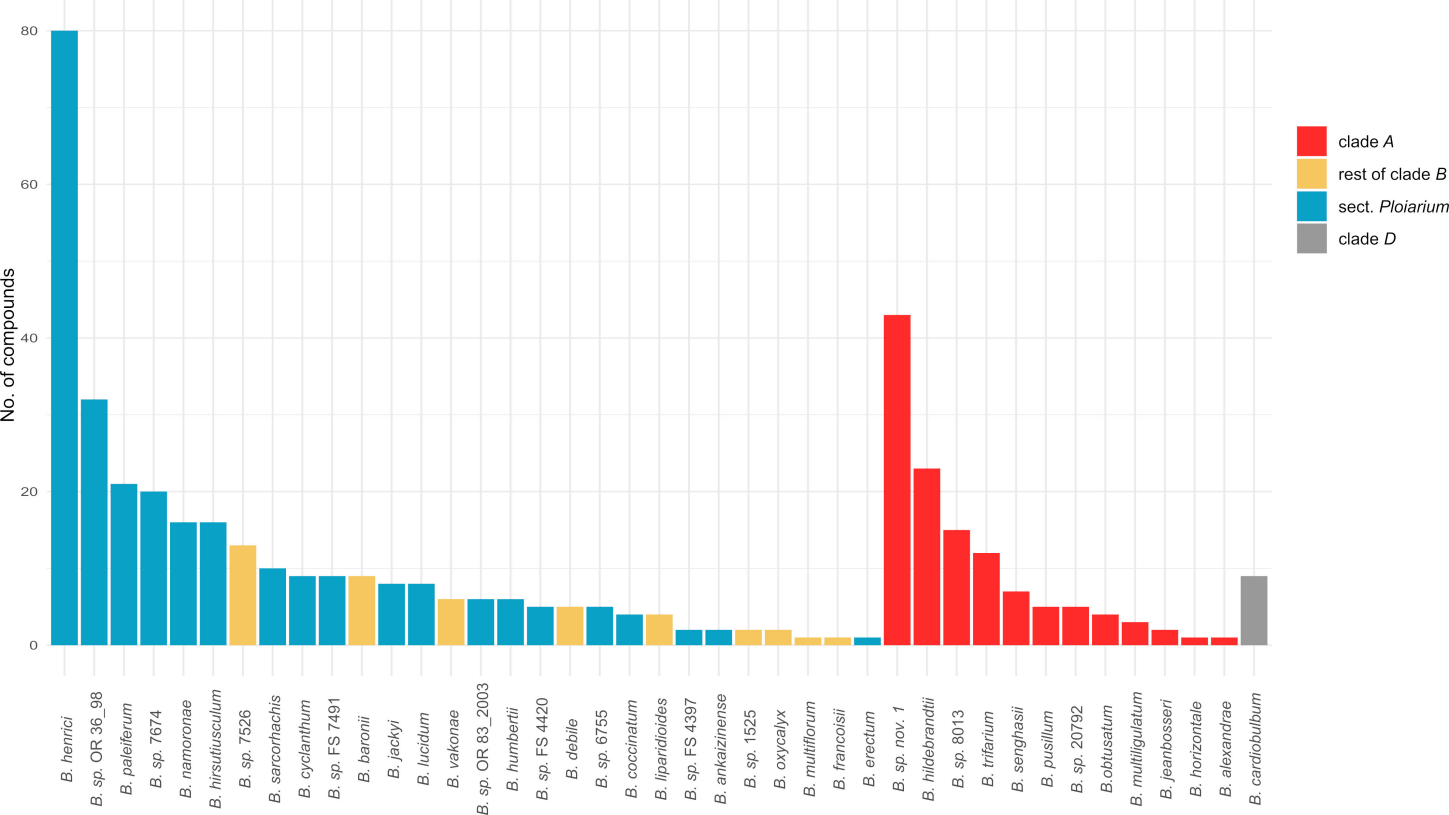
Number of floral volatile organic compounds (VOCs) per species of Malagasy *Bulbophyllum* (*n* = 41 in total; see also **Supplementary Table 1**). Colours represent clade/section memberships [i.e. clade *A*, rest of clade *B* (without sect. *Ploiarium*), sect. *Ploiarium*, and clade *D*].

Across the 41 species, we detected a total of 297 VOCs (**Supplementary Dataset S1**), most of which (225/*c*. 76%) were species-specific. Overall, 117, 226 and 10 compounds were found across species of clade *A* (12 spp.), clade *B* (28 spp.) and clade *D* (only *B. cardiobulbum*), respectively. Of the 226 compounds detected in clade *B*, 184 were only observed in members of sect. *Ploiarium* (19 spp.) (**Supplementary Dataset S1**). Of the 41 species, 30 had scent profiles consisting of only one (5 spp.) or fewer than 10 compounds, followed by six species with 10–20 compounds and only five with more than 20 compounds (**Figure 1**). Amongst the latter, *B.* sp. *nov.* 1 (sect. *Calamaria*/clade *A*) and *B. henrici* (sect. *Ploiarium*) emitted the most diverse scents with 43 and 80 compounds, respectively (**Figure 1**). There was no significant difference in the number of compounds between species of clades *A* (mean/median: 10.1/5; min/max: 1/43) vs *B* (10.8/6; 1/80; Wilcoxon test: *P* = 0.71), nor between those of clade *A*, sect. *Ploiarium* of clade *B* (13.7/8; 1/80), and the rest of clade *B* (4.78/4; 1/13; Kruskal–Wallis test: *P* = 0.12). Among the 41 species, (*E*)-β-caryophyllene was the most frequently shared compound (10 spp.), followed by (*E*,*E*)-α-farnesene (7 spp.) and (*E*)-β-ocimene (6 spp.), all of which are terpenoids (**Supplementary Dataset S1**).

**Figure 2:**
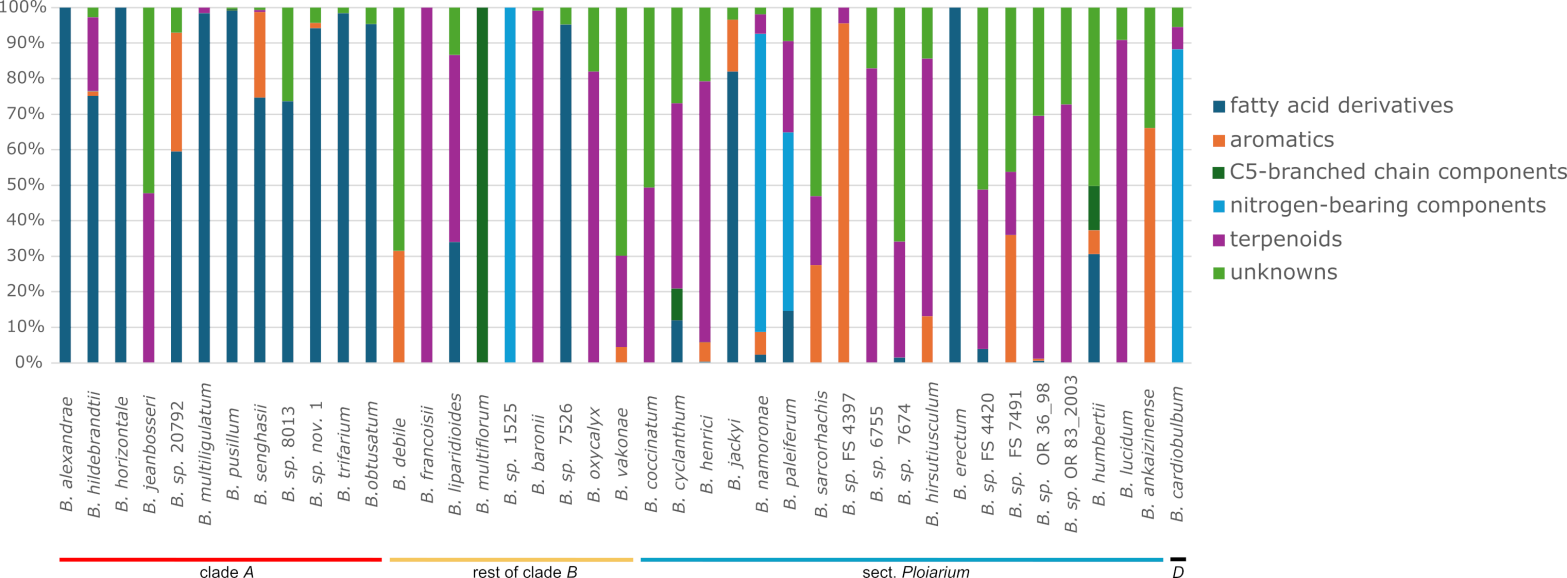
Relative proportion of six different chemical compound classes in the scent profiles of each of the 41 species of Malagasy *Bulbophyllum* included in this study. Each bar represents the scent profile of a single species, with colours indicating the percentage of compounds belonging to each class. Species clade/section memberships are indicated at the bottom.

Of the 297 VOCs detected, 152 could be identified and categorized into five chemical classes, including (i) terpenoids (71); (ii) fatty acid derivatives (56); (iii) aromatic compounds (benzenoids + phenylpropanoids) (16); (iv) nitrogenous (N-bearing) compounds (5); and (v) C5-branched chain compounds (4). **Table 1** shows the mean and median relative amounts of the dominant VOCs in different scent profile classes across different sections and clades. As further summarized in **Figure 2**, fatty acid derivatives were present in high relative amounts in all species of clade *A*, excepting one (*B. jeanbosseri*) that did not release such compounds at all, but only terpenoids and unknowns (‘UNK’). By contrast, fatty acid derivatives were rarely predominant in non-clade *A* species [i.e. *B.* sp. 7526 (rest of clade *B*), *B. erectum*, *B. jackyi* (both sect. *Ploiarium* of clade *B*)]. The chemical class profiles of species of sect. *Ploiarium* and non-*Ploiarium* species of clade *B* were largely similar and were mainly dominated by terpenoids (7 and 4 spp., respectively) and unknowns (6 and 2 spp., respectively), and to a lesser extent by C5-branched chain compounds (2 and 1 spp., respectively). Furthermore, two species of sect. *Ploiarium* (B. sp. FS 4397 and *B. ankaizinense*) were dominated by aromatic compounds, and one non-*Ploiarium* species of clade *B* (B. sp. 1525) by N-bearing compounds. The latter were also predominant in the clade *D* species *B. cardiobulbum* (**Figure 2**; see also below).

**Table 1:** Main volatile organic compounds (VOCs; sorted by chemical class) identified in the floral scents of 41 study species of Malagasy Bulbophyllum and their relative contribution (in %) to the scent across all species of clade A, rest of clade B (without sect. Ploiarium), and sect. Ploiarium, respectively (as mean and median values), plus a single species of clade D (B. cardiobulbum). Compounds with less than 1% contribution in one or more species per clade/section were subsumed within a given chemical class. See **Supplementary Dataset S1** (separate Excel file) for the absolute amounts of all 297 VOCs found in this study.

Chemical class/	Clade *A*		Rest of clade *B*		Sect. *Ploiarium*		Clade *D*
compound ^a^	(12 spp.)		(9 spp.)		(19 spp.)		(1 sp.)
	Mean	Median	Mean	Median	Mean	Median	
Fatty acid derivatives							
1-Decanol*	7.57	0	0	0	0.05	0	0
1-Dodecanol*	0.11	0	0	0	0	0	0
1-Octanol*	0.09	0	0	0	0	0	0
1-Octen-3-ol*	0	0	3.79	0	0.86	0	0
3-Octanone*	0	0	0	0	0.16	0	0
9-epi-(E)-Caryophyllene	0	0	0	0	0.26	0	0
Decanoic acid*	0.15	0	0	0	0.17	0	0
Dodecanal*	0.03	0	0	0	1.61	0	0
Dodecyl acetate*	6.21	0	0	0	0	0	0
Ethyl decanoate*	1.99	0	0	0	0	0	0
Ethyl dodecanoate*	5.64	0	0	0	0	0	0
Ethyl undecanoate	0.32	0	0	0	0	0	0
Heptadecene isomer	0.02	0	1.4	0	0	0	0
Hexanal*	0.53	0	0	0	0	0	0
Heptanoic acid*	0	0	0	0	5.26	0	0
Hexanoic acid*	0.35	0	0	0	0	0	0
Nonanoic acid*	0.3	0	0	0	0.04	0	0
Octanoic acid*	8.64	0	0	0	4.15	0	0
Pentadecadiene isomer	4.94	0	0	0	0	0	0
Pentadecane*	0.98	0	2.05	0	0.57	0	0
Pentadecene isomer 1	0.07	0	6.7	0	0	0	0
Pentadecene isomer 2	0.01	0	0.15	0	0	0	0
Tetradecane*	0.12	0	0	0	0	0	0
Tridecane*	23.02	0	0	0	0	0	0
Tridecene isomer 2	1.75	0	0	0	0	0	0
Undecane*	17.18	0	0	0	0	0	0
+ 30 compounds < 1%	0.64	0.33	0.29	0	0.09	0	0
Aromatics							
2,4-Dimethylbenzaldehyde	0	0	0.5	0	0	0	0
2-Phenylethanol*	0	0	0	0	1.56	0	0
3,5-Dimethoxytoluene*	0	0	0	0	0.63	0	0
Anethol*	0	0	3.5	0	0	0	0
Anisole*	0	0	0	0	5.17	0	0
Benzyl acetate*	0	0	0	0	1.55	0	0
Benzyl isovalerate	0	0	0	0	3.48	0	0
Methyl 2-hydroxy-4/5/or 6-methylbenzoate	4.8	0	0	0	0.74	0	0
Methyl salicylate*	0.08	0	0	0	1.11	0	0
Methyl vanillate	0.11	0	0	0	0	0	0
+ 6 aromatics < 1%	0.02	0	0	0	0.08	0	0
C5-branched chain compounds							
3-Methyl-1-butanol*	0	0	11.11	0	0	0	0
3-Methylbutanoic acid*	0	0	0	0	1.12	0	0
+ 2 compounds < 1%	0.05	0	0	0	0	0	0
Nitrogen-bearing compounds							
2-Aminobenzaldehyde*	0	0	0.27	0	0.24	0	0
Indole*	0	0	0	0	4.74	0	0
Methyl anthranilate*	0	0	0	0	0.36	0	0
N,N-Dimethylleucine O-methyl ester	0	0	0	0	0	0	88.33
N-Formyl-2-aminobenzaldehyde	0	0	10.84	0	1.71	0	0
Terpenoids							
(E)-Nerolidol*	0	0	0	0	0.55	0	0
(E)-α-Bergamotene	0	0	0	0	0.12	0	0
(E)-β-Caryophyllene	0	0	0	0	7	0.47	0
(E)-β-Farnesene*	0	0	0	0	1.1	0	0
(E)-β-Ocimene*	0.16	0	0.13	0	6.3	0	0
(E,E)-2,6-Dimethyl-1,3,5,7-octatetraene	0.05	0	0	0	3.38	0	0
(E,E)-2,6-Dimethyl-3,5,7-octatriene-2-ol	0	0	0	0	0.1	0	0
(E,E)-α-Farnesene*	0	0	0	0	5.5	0	0
(E,Z)-2,6-Dimethyl-1,3,5,7-octatetraene	0	0	0	0	0.05	0	0
(Z)-Linalool oxide furanoid*	0	0	0.2	0	0	0	0
(Z)-Linalool oxide pyranoid*	0	0	0.48	0	0	0	0
(Z,E)-α-Farnesene	0	0	0	0	0.17	0	0
1,3,8-p-Menthatriene	0	0	0	0	0.07	0	0
3-Pentanone	0	0	0	0	0.08	0	0
4-Oxoisophorone*	0	0	1.12	0	0	0	0
6-Methyl-5-hepten-2-one*	0	0	5.84	0	1.87	0	0
Caryophyllene oxide*	0	0	0	0	1	0	0
Geraniol*	4.11	0	9.16	0	0	0	0
Geranyl acetate*	0.24	0	0.11	0	0	0	0
Germacrene D*	0	0	11.11	0	0.01	0	0
Hexahydrofarnesylacetone	0.01	0	0	0	0.73	0	0
Hotrienol	0	0	0.37	0	0	0	0
Humulene epoxide isomer	0	0	0	0	0.15	0	0
Lavandulol	0.13	0	0	0	0	0	0
Limonene*	0	0	0	0	1.84	0	0
Linalool*	0	0	9.65	0	3.83	0	0
Nerol*	0.9	0	0	0	0	0	0
Neryl acetate*	0.1	0	0	0	0	0	0
p-Cymene + unknown	0	0	0	0	0.49	0	0
ß-Bourbonene*	0	0	0	0	0.19	0	0
Valencene	0	0	0	0	0	0	1.37
Verbenone*	0	0	1.73	0	0	0	0
α-Caryophyllene*	0	0	0	0	1.4	0	0
α-Pinene*	0	0	0	0	0.17	0	0
α-Selinene*	0	0	0	0	0.22	0	2.98
β-Bisabolene*	0	0	0	0	0.18	0	0
β-Myrcene*	0	0	0	0	0.23	0	0
β-Selinene*	0	0	0	0	0.01	0	1.84
γ-Elemene	0	0	0	0	0.14	0	0
+ 30 compounds < 1%	0.19	0	0.05	0	0.49	0	0
Unknowns	8.42	2.13	19.45	8.99	26.93	26.9	5.49

a Compounds identified based on synthetic compounds are marked by an asterisk. *Ploiarium*, respectively (as mean and median values), plus a single species of clade *D* (*B. cardiobulbum*). Compounds with less than 1% contribution in one or more species per clade/section were subsumed within a given chemical class. See **Supplementary Dataset S1** (separate Excel file) for the absolute amounts of all 297 VOCs found in this study.

### Multivariate analyses of floral scent compounds among species and clades/sections

**Figure 3:**
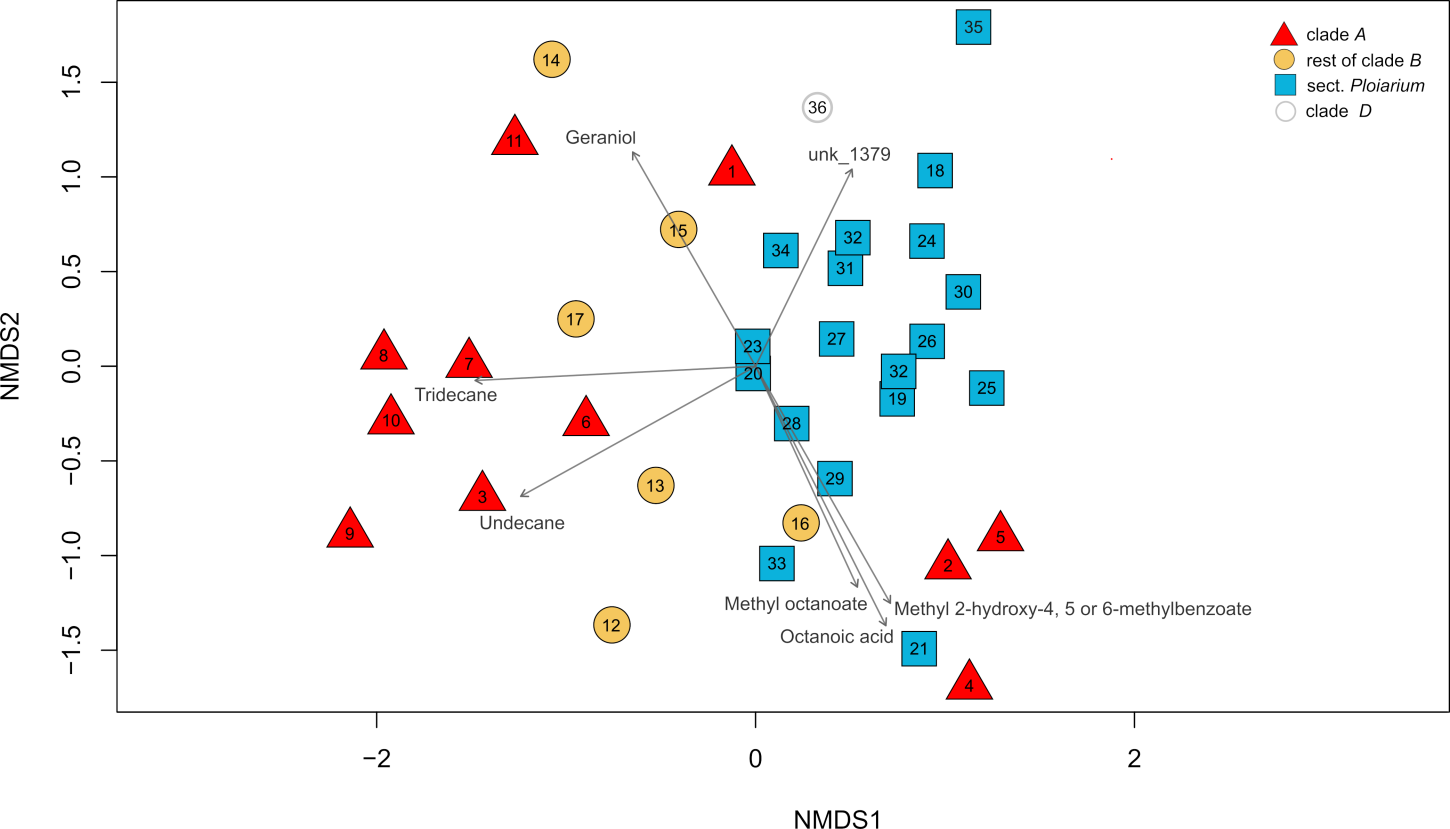
Nonmetric multidimensional scaling (NMDS) ordination plot based on a Bray–Curtis dissimilarity matrix of relative abundances of floral volatile organic compounds (VOCs) between 36 species of Malagasy *Bulbophyllum*. Sample symbols and colours represent clade/section memberships [i.e. clade *A*, rest of clade *B* (without sect. *Ploiarium*), sect. *Ploiarium*, and clade *D*] and their numbers refer to the respective species ([(1) B. hildebrandtii; (2) B. sp. 20792; (3) B. pusillum; (4) B. senghasii; (5) B. sp. 8013; (6) B. sp. nov. 1; (7) B. trifarium; (8) B. alexandrae; (9) B. horizontale; (10) B. multiligulatum; (11) B. jeanbosseri; (12) B. francoisii; (13) B. sp. 1525; (14) B. sp. 7619; (15) B. sp. 7433; (16) B. liparidioides; (17) B. sp. 7526; (18) B. coccinatum; (19) B. cyclanthum; (20) B. henrici; (21) B. jackyi; (22) B. namoronae; (23) B. paleiferum; (24) B. sarcorhachis; (25) B. sp. FS 4397; (26) B. sp. 6755; (27) B. sp. 7674; (28) B. sp. 7691; (29) B. sp. FS 4420; (30) B. sp. FS 7491; (31) B. sp. OR 36_98; (32) B. sp. OR 83_2003; (33) B. sp. ORCH000463; (34) B. sp. S 2704; (35) B. sp. S 7143; (36) B. cardiobulbum]). Arrows indicate the vectors of significantly (*P* < 0.05) associated scent compounds. See **Supplementary Figure 2** for the corresponding NMDS biplot of the full dataset of 41 species, i.e. with five outlier taxa included (see text for details).

The NMDS biplot, based on the relative abundances of VOCs of the 41 species (**Supplementary Figure S2**), largely failed to distinguish them, except for five highly divergent species (i.e. *B. debile*, *B. erectum, B. multiflorum*, *B. obtusatum*, *B. vakonae*; see also **Supplementary Dataset S1** for details). After excluding these ‘outliers’, the other species tended to group along the first axis (NMDS1) according to clade/section membership (i.e. clade *A*, sect. *Ploiarium*, rest of clade *B*); notably, however, three species of clade *A* (i.e. *B.* sp. 20792, *B. senghasii*, *B.* sp. 8015) showed greater similarity to those of sect. *Ploiarium* than to their closer relatives (**Figure 3**). Only seven VOCs were significantly (*P* < 0.05) correlated with the two axes, including tridecane/undecane (only clade *A*), geraniol (only clade *A* and non-*Ploiarium* spp. of clade *B*), UNK1379 (only sect. *Ploiarium*), as well as octanoic acid, methyl octanoate and an isomer of methyl 2-hydroxy methylbenzoate [methyl 2-hydroxy-4, 5 or 6-methylbenzoate] (only clade *A* and sect. *Ploiarium*).

Overall, PERMANOVA revealed a significant difference in the relative scent composition among the three groups (pseudo-*F*_(3, 32)_ = 1.52, *P* < 0.001; see also **Figure 2**), and the same was true for the dispersion (variability) of their scent profiles (PERMDISP: pseudo-*F*_(3, 32)_ = 65.29, *P* < 0.001). Pairwise PERMANOVA comparisons showed a significant difference in the relative scent composition between species of clade *A* and sect. *Ploiarium* (*P* = 0.006), but not between clade *A* and the rest of clade *B* or between sect. *Ploiarium* and the rest of clade *B* (both *P* > 0.68). Despite the significant global PERMDISP test, no pairwise comparison was significant (all *P* > 0.29), suggesting that the significant among-group differences observed are probably not due to within-group dispersion. As illustrated by the NMDS vectors (**Figure 3**), the scent profiles of clade *A* spp. were characterized by high relative amounts of fatty acid derivative alkanes and alkenes, such as undecane and tridecane (e.g. *B. multiligulatum, B. trifarium*), by ethyl dodecanoate and ethyl decanoate (*B. obtusatum*), or by octanoic acid (*B. senghasii*) (see also **Figures 2**, **3**; **Table 1** and **Supplementary Dataset S1**). Other species of clade *A* released high amounts of the C5-branched chain compound 2-methylbutanoic acid (*B. pusillum*) or high amounts of geraniol (terpenoid) and UNK1570 (*B. jeanbosseri*; **Supplementary Dataset S1**). The terpenoid-dominated scents of sect. *Ploiarium* species of clade *B* were characterized by high relative amounts of linalool, limonene, and (E)-β-caryophyllene (1 sp. each), (E)-β-ocimene (2 spp.), or (E)-β-caryophyllene and (*E*,*E*)-α-farnesene (2 spp.); other species of this section mainly released the aromatics benzyl isovalerate (*B*. *ankaizinense*) and anisole (*B.* sp. FS 4397), the N-bearing compounds indole (*B. namoronae*) and indole together with N-formyl-2-aminobenzaldehyde (*B. paleiferum*), and the fatty acid derivatives octanoic acid (*B. jackyi*) and hexanoic acid (*B*. *erectum*). Non-*Ploiarium* species of clade *B* emitted compounds from various classes, such as the terpenoids 6-methyl-5-hepten-2-one (*B. liparidioides*), linalool (*B. baronii*), geraniol (*B. oxycalyx*) and germacrene D (*B. francoisii*), the fatty acid derivative pentadecane (*B*. sp. 7526), or the N-bearing N-formyl-2-aminobenzaldehyde (*B.* sp. 1525). One species of this group exclusively released the C5-branched chain compound 3-methyl-1-butanol (*B. multiflorum*). Finally, the scent of *B. cardiobulbum* (clade *D*) was strongly dominated by N,N-dimethylleucine O-methyl ester, a compound not detected in any other species (**Table 1**; **Supplementary Table S2**; see also **Supplementary Methods S1**).

### Phylogenetic signal and models of floral scent evolution

Our estimate of phylogenetic signal for the relative abundance data of shared VOCs (59 in total) across the pruned 32-spp. phylogeny (**Supplementary Figure S1**) was lower than expected under the BM model of trait neutrality (*K*_mult_ = 0.233) but non-significant (*P* = 0.48). Hence, even though distant relatives appeared to resemble each other more (0 < *K*_mult_ < 1) than expected by chance, there was insufficient statistical evidence to firmly reject the null hypothesis of Brownian motion (e.g. Blomberg et al., 2003). By contrast, our PL analyses in rpanda clearly showed that a single-optimum OU model of constrained trait evolution provided a far better fit to the shared VOC data (lowest GIC value) than both the BM and EB models (**Table 2**). Our estimated parameter value for the strength of this OU model, *α*, was 2.05 and relatively large (≥ 2; Beaulieu et al., 2012), indicating a medium-to-strong attraction (‘pull’) of whole scent bouquet towards an adaptive optimum across the phylogeny (e.g. Voje and Hansen, 2013; O’Meara and Beaulieu, 2014). Accordingly, the corresponding estimate of phylogenetic half-life (*t*_1/2_) was 0.33 Myr.

**Table 2:** Evolutionary model fitting to the whole scent bouquet of 32 Malagasy Bulbophyllum species (59 volatile organic compounds, VOCs), using a penalized likelihood (PL) approach (Clavel et al., 2019), as implemented in RPANDA v.2.3. Each model (i.e. BM, Brownian motion; EB, early burst; OU, single-optimum Ornstein–Uhlenbeck) was evaluated over the time-calibrated maximum clade credibility (MCC) tree of Malagasy Bulbophyllum (Gamisch et al., 2021) pruned to these 32 species for which shared VOC data could be obtained in the present study (see also **Supplementary Figure S1**).

Model	Parameter	GIC	ΔGIC
BM	/	10806.786	26.99
EB	*r* = 0	10808.78	28.99
OU	*α* = 2.05	10779.79	0

*r*, EB parameter of exponential rate decrease; *α*, rate of adaptation parameter for OU; GIC, Generalized Information Criterion; ΔGIC, difference in GIG value between the best model and the model being compared. Note: all models (BM, EB, OU) estimate *R*, the multivariate counterpart of *σ*^2^⁠, the diffusion parameter of the univariate BM model, while the EB and OU models estimate an extra parameter (*r* and *α*, respectively). Following Clavel et al. (2019), only the latter are shown here, while the Brownian parameters are given in the high-dimensional *R* matrix (available upon request). Each model (i.e. BM, Brownian motion; EB, early burst; OU, single-optimum Ornstein–Uhlenbeck) was evaluated over the time-calibrated maximum clade credibility (MCC) tree of Malagasy *Bulbophyllum* (Gamisch et al., 2021) pruned to these 32 species for which shared VOC data could be obtained in the present study (see also **Supplementary Figure S1**).

Finally, fitting each of the three models (BM, EB, OU) to each of the 59 VOCs separately in geiger revealed that most of them (41) best-fitted a single-optimum OU process and far less frequently an EB (7) or BM (4) model (**Supplementary Table 2**). In seven instances, our test could not distinguish between the EB and OU models, which received the same fit (ΔAICc = 0). However, even in instances where one model was preferred, others had essentially equivalent fit (ΔAICc < 2; **Supplementary Table 2**), indicating high uncertainty in model selection, especially between EB and OU. Among those compounds that unambiguously best fitted the OU model, the corresponding *α* parameters varied widely, ranging from 1.67 to 10.0 (**Supplementary Table 2**), implying different levels of trait-specific rates at which species adapted towards their present optima (Beaulieu et al., 2012; Voje and Hansen, 2013; Cooper et al., 2016). In two VOCs (i.e. linalool and geranyl acetate), the estimated *α* parameter was at the upper boundary of the search interval (*α* = 10.0), suggesting a very strong pull towards the optimum, which may reflect either true strong stabilizing selection or at least little phylogenetic influence on these particular compounds (see also Cooper et al., 2016).

## Discussion

### General patterns of floral scent variation and compound identification

This study analysed, for the first time, scent variation and mode of evolution in Malagasy species of the pantropical orchid genus *Bulbophyllum*. Our analysis included 41 species of this large Mid-to-Late Miocene (≤ 12.7 Ma) island radiation (*c*. 210 spp.; Gamisch et al., 2021) and detected a total of 297 VOCs (**Supplementary Dataset S1**). Overall, the number of floral scent compounds varied considerably among species (range: 1–80 VOCs; **Figure 1**), which is consistent with similar, albeit rare surveys in other tropical orchid genera (e.g. *Catasetum*: Milet-Pinheiro and Gerlach, 2017; see also Van der Niet et al., 2010; Hetherington-Rauth and Ramírez, 2016). Notably, most of the 297 VOCs detected in Malagasy *Bulbophyllum* were species-specific (225/*c*. 76%; **Supplementary Dataset S1**). We can only speculate that this high floral scent specificity reflects a high degree of specialized (fly) pollination (Humeau et al., 2011; Hermans et al., 2021; Pailler and Baider, 2020; see also Raguso, 2020), which in turn is often considered an important, if not a key factor for promoting species diversification and rapid radiation in many orchid lineages (e.g. Tremblay, 1992; Breitkopf et al., 2015; Ray and Gillett-Kaufman, 2022; Ackerman et al., 2023; see also further below).

Many of the 147 (out of 297) VOCs that we could identify and classify (**Figure 2**; **Table 1** and **Supplementary Figure S2**) are known to be physiologically and/or behaviorally active scent components of orchids with different pollination strategies/pollinators (reviewed in Perkins et al., 2023). Examples include fatty acid derivative compounds (e.g. *n*-pentadecane in sexually deceptive *Ophrys insectifera* pollinated by male wasps), terpenoids [e.g. (*E*)-β-farnesene in hoverfly pollinated *Cypripedium subtropicum*; 4-oxoisophorone in mainly bee-pollinated *C. calceolus*], aromatics (e.g. 2-phenylethanol and 2-phenylethyl acetate in moth and butterfly pollinated *Gymnadenia conopsea*), and nitrogenous compounds (e.g. indole in perfume orchids attractive to euglossine male bees). In addition, we found, geraniol and nerol, two very common floral volatiles in orchids that, for instance, are attractive to male euglossine bees (Perkins et al., 2023). We also found limonene, (*E*)-β-ocimene, β-myrcene and linalool, which are even the most common floral volatiles in angiosperms (Knudsen et al., 2006) and are involved as chemical mediators in various orchid pollination systems (Dobson, 2006; Dötterl and Gershenzon, 2023; Liu et al., 2024).

The compound most widely shared among our 41 Malagasy *Bulbophyllum* study species, (*E*)-β-caryophyllene (10 spp.), has previously been shown (in a blend with other compounds) to be attractive to pollinating ants in *Chamorchis alpina* and to pollinating hoverflies in *Cypripedium subtropicum* (Perkins et al., 2023); however, this compound could also play a role in plant defense to herbivores and/or pathogens, as is known from other angiosperm families (e.g. Huang et al., 2012; Bouwmeester et al., 2019). Our analysis did not detect compounds reported as attractants and rewards in Southeast Asian *Bulbophyllum* species pollinated by tephritid fruit flies (e.g. raspberry ketone, zingerone, zingerol, anisyl acetone, rhododendrol, and methyl eugenol; Tan and Nishida, 2000, Tan and Nishida, 2007; Tan et al., 2002, Tan et al., 2006, Tan et al., 2023; Nishida et al., 2004). This likely reflects the very specialized floral-pollinator adaptation in these Southeast Asian species, with male fruit flies using these floral scent compounds as pheromone precursors to attract females, or to boost their defence system (Tan and Nishida, 2000; Tan et al., 2002). However, we found several compounds that were recently also identified among five (mainly Asian) *Bulbophyllum* species of the ‘*Cirrhopetalum* alliance’ (i.e. *a*-copaene, 3-methyl-1-butanol, 6-methyl-5-hepten-2-one, limonene, caryophyllene, pentadecane and undecane; Davies et al., 2025); however, observations of insect visitors are only available for one of these species, i.e. *B. bicolor*, whose sole effective pollinator is probably a dung-breeding muscoid fly (Hu et al., 2017). Perhaps not surprisingly, some of our identified VOCs have also previously been detected in accessions of the sapromyiophilous *B. variegatum* (sect. *Alcistachys*) from La Réunion (i.e. β-elemene, (*E*)-β-caryophyllene, *a*-caryophyllene, and indole; Humeau et al., 2011). Unfortunately, neither this species nor other members of sect. Alcistachys (*clade A*; Gamisch et al., 2021) were included in the present study. Nevertheless, fetid-smelling indole, as typical for *B. variegatum*, was also the most abundant compound in two of our study species of sect. *Ploiarium* (i.e. *B. namoronae* and *B. paleiferum*), which also emitted other N-bearing compounds, such as 2-aminobenzaldehyde and N-formyl-2-aminobenzaldehyde. Both latter compounds derive from indole (Spiteller and Steglich, 2001), but, to our knowledge, do not occur in sapromyiophilous plant species (Jürgens et al., 2013), but rather in rewarding taxa pollinated by bees or moths (Joulain, 1987; Dötterl and Gershenzon, 2023). It remains to be clarified whether *B. namoronae* and *B. paleiferum* have a similar or different pollination system than *B. variegatum.* 2-Aminobenzaldehyde and N-formyl-2-aminobenzaldehyde were even the exclusive compounds of a still undescribed species of sect. *Elasmotopus* (*B.* sp. 1525). Notably, our survey revealed another N-bearing compound, namely N,N-dimethylleucine O-methyl ester, which was emitted exclusively and in high relative amounts by *B. cardiobulbum* (clade *D*/sect. *Inversiflora*). To our knowledge, this compound is documented here for the first time as a floral scent, but is also known from other natural sources, such as fig leaves (Kaiser, 1986). No data on the biosynthesis of this compound are currently available; however, it is very likely that certain enzymes (e.g. leucine carboxyl methyltransferases) methylate the amino acid leucine using *S*-adenosyl-_L_-methionine (SAM) as a methyl source (see also Creighton et al., 2022; Li et al., 2024). Further research is required to elucidate the biosynthesis of N,N-dimethylleucine O-methyl ester and also to examine whether any of the three N-bearing compounds mentioned above are involved in attracting pollinators. In particular, it would be interesting to know whether N,N-dimethylleucine O-methyl ester is responsible for the attraction of tiny (*c*. 1.7 mm long) female flies of hitherto unknown species of the genus *Arcuator* Saprosky (Chloropidae), which have been observed as potential pollinators of *B. cardiobulbum* (Hermans et al., 2021). However, there is already evidence that at least 2-aminobenzaldehyde elicits physiological responses in the antennae of some pollinating insects (Dötterl and Gershenzon, 2023).

### Selectively constrained evolution of whole scent bouquets and most single compounds

According to our multivariate statistical analyses (e.g. **Figure 3**), floral scents of the different species tend to cluster according to section/clade membership; however, we also observed some overlap between these groups, suggesting that floral scent in Malagasy *Bulbophyllum* may have evolved to some extent independently of phylogenetic history, which is indeed supported by the PCM-based analyses of phylogenetic signal. Specifically, for the relative abundance data of shared VOCs (59 in total) across the pruned 32-spp. phylogeny (**Supplementary Figure S1**), our estimate of phylogenetic signal was lower than expected under the BM model of trait neutrality (*K*_mult_ = 0.233). However, we caution that the associated *P* value was non-significant (0.48). Therefore, we cannot exclude the possibility that our estimated value of *K*_mult_, even though low (< 1), is *not* significantly greater than would be expected if trait variation were randomly distributed across the phylogeny (Revell, 2024). Nevertheless, the low level of phylogenetic signal detected herein could mean high evolutionary trait lability (e.g. Feulner et al., 2014; Prieto-Benítez et al., 2016; Heiduk et al., 2017; Liu et al., 2024), with the *K*_mult_ value < 1 indicating that distantly related species resemble each other more in their whole scent bouquets than expected under a purely neutral, rate-constant (BM) model, hence obliterating the effect of their phylogenetic history (Blomberg et al., 2003). In fact, weak (or non-significant) phylogenetic signals for whole floral scent have previously been reported (Farré-Armengol et al., 2015; Prieto-Benítez et al., 2016; Heiduk et al., 2017; Wang et al., 2019; Milet‐Pinheiro et al., 2021; Rabeschini et al., 2021; Liu et al., 2024).

However, to our knowledge, no study to date has employed explicit PCM-based modelling to test the hypothesis that such a weak phylogenetic signal of floral scent could also be associated with a constrained OU model of trait evolution (Blomberg et al., 2003; Lavin et al., 2008; Münkemüller et al., 2012). In fact, our respective PL model testing in rpanda revealed that the evolution of whole scent bouquet across the 32-spp. phylogeny of Malagasy *Bulbophyllum* best fitted a single-optimum OU process (**Table 2**). Moreover, the estimate of the associated *α* parameter was relatively large (2.05; i.e. ≥ 2), indicating a medium to strong pull of this highly complex trait towards an optimal value (e.g. Hansen, 2014; O’Meara and Beaulieu, 2014). Many studies have highlighted the fundamental role of scent in *Bulbophyllum* in luring insects to the flower and guiding them towards the column for pollinia removal or deposition (e.g. Nakahira et al., 2018; Wiśniewska et al., 2023; see also Introduction). Our modelling results therefore support the hypothesis that whole scent evolution in Malagasy *Bulbophyllum* is constrained, possibly because of strong pollinator-mediated stabilizing/directional selection (e.g. Joffard et al., 2020; De Cauwer et al., 2025).

Phylogenetic signals in floral scent have been found more frequently when analysing single compounds, rather than whole bouquets (Llusià et al., 2010; Farré-Armengol et al., 2015; Prieto-Benítez et al., 2016; Liu et al., 2024), suggesting that different VOCs could reliably reflect phylogenetic relatedness by potentially evolving individually rather than in concert as an integrated trait (Schwery et al., 2023). For example, Joffard et al. (2020) detected relatively high phylogenetic signals in whole scent bouquets of sexually deceptive *Pseudophrys* species but also noted that different classes of compounds showed different levels of evolutionary lability and conservativeness, possibly reflecting their idiosyncratic roles in plant–pollinator interactions (see also Liu et al., 2024, for similar findings in Catasetinae and Stanhopeinae). Our trait modelling in geiger showed that the evolution of single VOCs in Malagasy *Bulbophyllum* followed, at least in part, different trajectories (**Supplementary Table 2**). Although most of them (41 out of 59) still best-fitted a single-optimum OU model, a few others apparently evolved according to an EB (7) or BM (4) process, although with some model selection uncertainty. Furthermore, even for the clearly OU-constrained VOCs, *α* parameter values varied widely (range: 1.67–10.0; **Supplementary Table 2**), implying that these compounds had evolved at different rates toward their current adaptive optima (Beaulieu et al., 2012; Voje and Hansen, 2013; Cooper et al., 2016). Overall, this suggests that different compounds could have evolved under different levels of constraint, perhaps due to their varied attractiveness to pollinators and/or their different functional roles (e.g. pollinator attraction vs florivore defence; Nakahira et al., 2018; Wiśniewska et al., 2023; see also Kite and Hetterscheid, 2017).

## Conclusions

Our analyses revealed high specificity and variability in the floral scent composition of Malagasy *Bulbophyllum* species, both in terms of semi-quantity and quality of VOCs, and also led to the first identification of N,N-dimethylleucine O-methyl ester as a floral scent compound. This high diversity and specificity of floral compounds, as well as the fact that some of them have previously been shown to be physiologically and/or behaviorally active during orchid pollination, suggest that scent has an important role in the pollinator attraction of Malagasy *Bulbopyllum*, and thus might be under selection by the group’s main pollinators (small Dipteran flies). The results of our PCM-based modelling approach further support this hypothesis, as a purely neutral (BM) process of whole scent bouquet evolution was clearly rejected in favour of a constrained (OU) model that accounts for the effect of stabilizing/directional selection. Furthermore, our model-based results indicate that individual VOCs have evolved, at least to some extent, along different trajectories, likely due to their different roles in pollinator attraction and/or other plant functions (e.g. florivore defence).

Clearly, however, further PCM-studies based on expanded interspecific floral scent data, and in combination with comprehensive information on pollinator species, are required to achieve a better understanding of the macroevolutionary role of VOCs in driving species diversification and plant–pollinator interactions in Malagasy *Bulbophyllum*. That said, any answer to the relative importance of floral scent in promoting speciation in this tropical orchid lineage will probably only emerge after detailed pollination-ecological and chemical analyses at the population level of well-supported sister species (e.g. *B. bicoloratum*/*B. occultum*; Jaros et al., 2016; Gamisch et al., 2021), i.e. relative to other putative prezygotic RI traits [phenological, floral (colour, shape, lip microstructure) etc.], and ideally in combination with genome-wide association studies (e.g. Hsu et al., 2022).

## Data Availability

The original contributions presented in the study are included in the article/**Supplementary Material**. Further inquiries can be directed to the corresponding author.
