# Supplementary material for: Diverse floral scents in Malagasy Bulbophyllum evolve in a bounded fashion
Source: Front Plant Sci. 2026 Mar 24;17:1785538. doi: 10.3389/fpls.2026.1785538 (PMC13055554; doi:10.3389/fpls.2026.1785538)
Supplement: Supplementary file 1 [file DataSheet1.docx]

**Supporting Information**

**Article title**: Diverse floral scents in Malagasy Bulbophyllum evolve in a bounded fashion

**Authors**: Silvia Artuso, Alexander Gamisch, Anton Sieder, Roman Fuchs, Sven Gindorf, Mario Schubert, Stefan Dötterl and Hans Peter Comes

The following Supporting Information is available for this article:

**Fig. S1** Phylogeny of 32 species included in the scent analysis, generated by pruning the time-calibrated maximum clade credibility (MCC) tree of Malagasy Bulbophyllum (Gamisch et al., 2021), based on concatenated nuclear and plastid DNA sequence data.

**Fig. S2** Nonmetric multidimensional scaling (NMDS) based on a Bray–Curtis dissimilarity matrix of relative abundances of floral volatile organic compounds (VOCs) between all 41 species of Malagasy *Bulbophyllum* of this study (i.e. with five outlier taxa included).

**Table S1** Sample and voucher information for the 41 species and 50 accessions of Malagasy *Bulbophyllum* included in this study (nine sections; clades *A*, *B*, *D*; *sensu* Gamisch *et al*., 2021) plus details on the scent sampling per accession [date, duration, site, collector(s)].

**Table S2** Evolutionary model fitting (BM, EB, OU) to each of the 59 floral volatile organic compounds (VOCs) shared among 32 species of Malagasy *Bulbophyllum*, using geiger.

**Methods S1** Identification of a novel floral scent compound, N,N-dimethylleucine O-methyl ester, using Nuclear Magnetic Resonance (NMR) spectroscopy.

**Dataset S1** (separate Excel file) List of all 297 volatile organic compounds (VOCs) detected in the 41 study species of Malagasy *Bulbophyllum*, including absolute amounts (peak areas, total ion currents). If more than one accession per species was analysed, absolute amounts were averaged across samples.


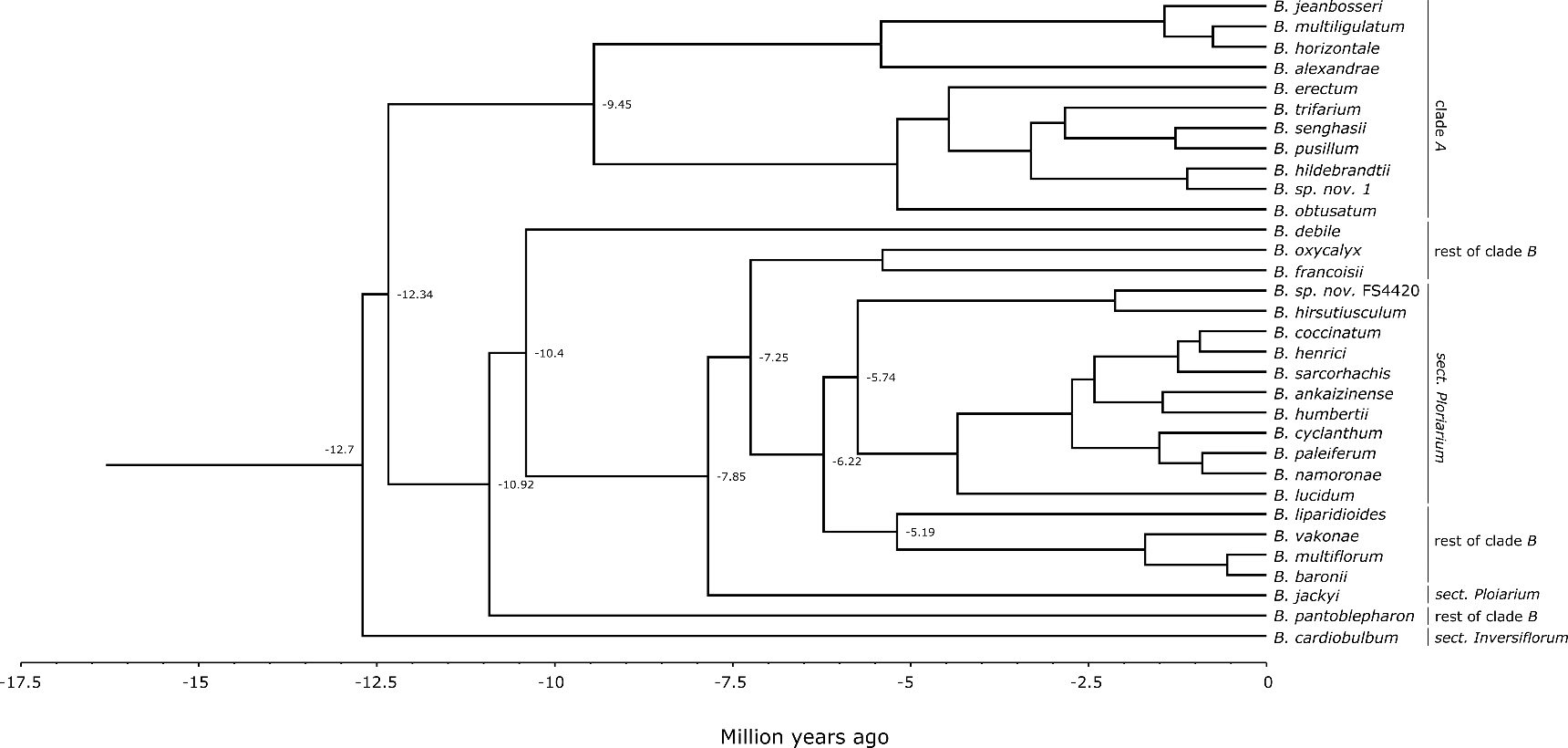


**Fig. S1** Phylogeny of 32 species included in the scent analysis, generated by pruning the time-calibrated maximum clade credibility (MCC) tree of Malagasy *Bulbophyllum* (Gamisch *et al.*, 2021), based on concatenated DNA sequence data of three nuclear (nrITS, *PI, Xdh*) and five plastid gene regions (*atp*I–*atp*H, *ycf1*, *mat*K, *trn*D*–trn*E, *psb*A*–trn*H). This pruned 32 spp.-phylogeny was generated using the function *drop.tips* in the R package phytools v.2.1-1.

**
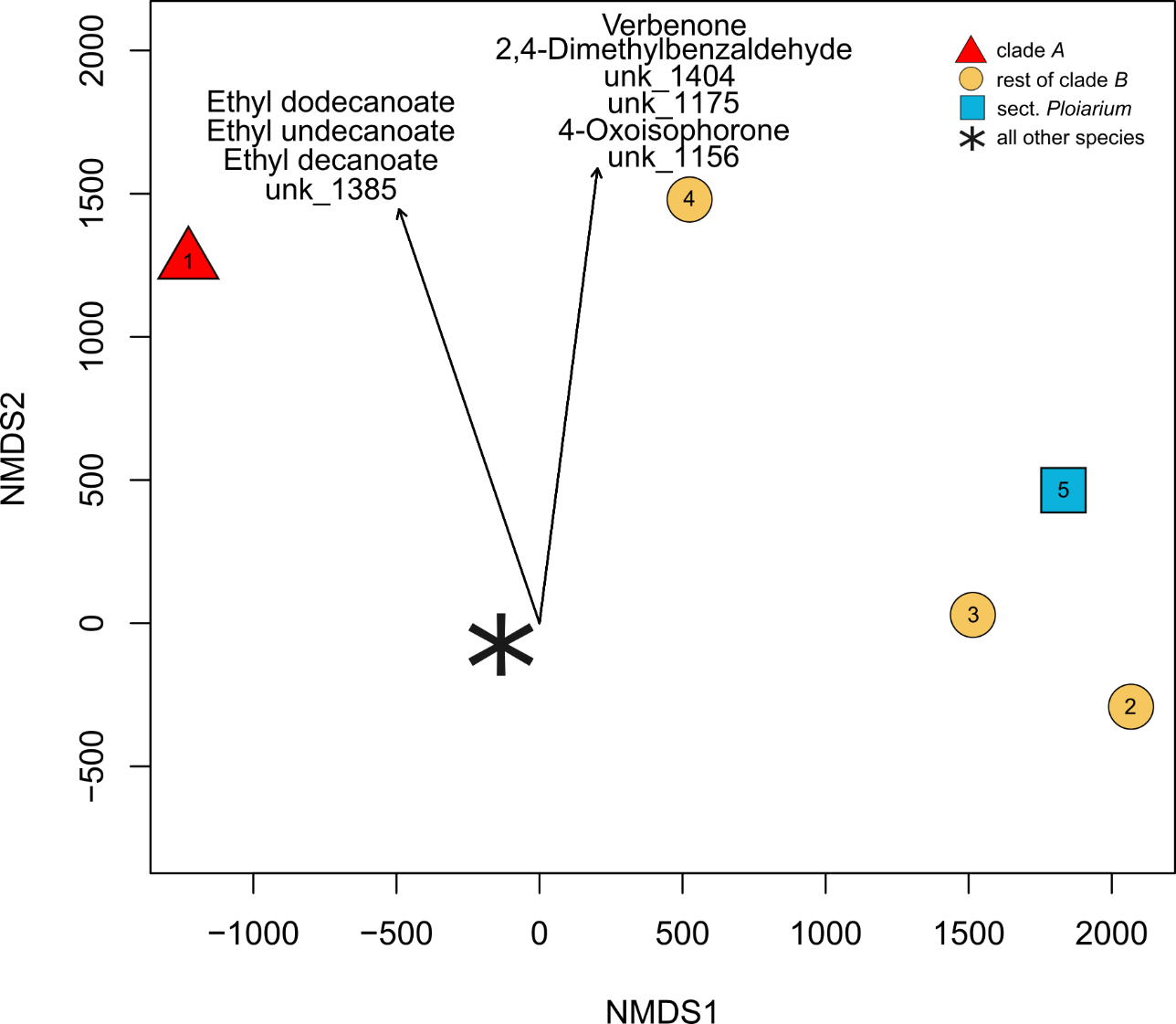
**

**Fig. S2** Nonmetric multidimensional scaling (NMDS) based on a Bray–Curtis dissimilarity matrix of relative abundances of floral volatile organic compounds (VOCs) between all 41 Malagasy Bulbophyllum species of this study. Sample symbols and colours represent clade/section memberships of five outlier species [i.e. clade A, rest of clade B (without sect. Ploiarium), sect. Ploiarium] with highly distinct scent profiles, and which are separated in the graph; all other 36 species, including one of clade D) clustered in the center (marked by an asterisk) and thus remained indistinguishable. Arrows indicate the vectors of significantly (P < 0.05) associated scent compounds. Code number identification of outlier species: 1, B. obtusatum; 2, B. debile; 3, B. melleum; 4, B. sp. 7664; 5, B. sp. FS 1021.

**Table S1** Sample and voucher information for the 41 species and 50 accessions of Malagasy *Bulbophyllum* included in this study (nine sections; clades *A*, *B*, *D*; *sensu* Gamisch *et al*., 2021) plus details on the scent sampling per accession [date, duration, site, collector(s)]. The 32 species included in the phylogenetic comparative analysis of scent variation (Fig. S1; Table 2) are marked by an asterisk.

| Species | | Section (clade) | Voucher no. | Scent sampling | | | | |
| --- | --- | --- | --- | --- | --- | --- | --- | --- |
|  |  |  |  |  | Date | Duration  (min) | Site ^a^ | Collector(s) ^b^ |
| ******* | *B. alexandrae* Schltr. | *Kainochilus* (*A*) | 7767 |  | 01/03/2018 | 20 | HBV | AS |
| ******* | *B. ankaizinense* Schltr. | *Ploiarium* (*B*) | S 7143 |  | 17/07/2017 | 15 | HBV | AS |
| ******* | *B. baronii* Ridl. | *Loxosepalum* (*B*) | 7433 |  | 23/01/2018 | 5 | M | AG, AS, DP |
| ******* | *B. cardiobulbum* Bosser | *Inversiflora* (*D*) | 7439 |  | 23/01/2018 | 15 | M | AG, AS, DP |
| ******* | *B. coccinatum* H.Perrier | *Ploiarium* (*B*) | FS 5961 |  | 21/07/2018 | 20 | HBV | AS |
| ******* | *B. cyclanthum* Schltr. | *Ploiarium* (*B*) | 7746 |  | 13/06/2019 | 150 | HBV | AS |
| ******* | *B. debile* Bosser | *Lichenophylax* (*B*) | 7661 |  | 05/02/2018 | 30 | M | AG, AS, DP |
| ******* | *B. erectum* Thouars | *Ploiarium* (*B*) | FS 1021 |  | 31/07/2018 | 20 | HBV | AS |
| ******* | *B. francoisii* H.Perrier | *Loxosepalum* (*B*) | 7718 |  | 25/01/2019 | 150 | HBV | AS |
| ******* | *B. henrici* Schltr. (1) | *Ploiarium* (*B*) | ORCH940162 |  | 29/06/2017 | 10 | HBV | AS |
|  | *–* (2) |  | 3814 |  | 02/04/2018 | 20 | HBV | AS |
| ******* | *B. hildebrandtii* Rchb.f. (1) | *Calamaria* (*A*) | FS 7110 |  | 29/06/2017 | 10 | HBV | AS |
|  | *–* (2) |  | 7110 |  | 27/02/2019 | 150 | HBV | AS |
| ******* | *B. hirsutiusculum* H.Perrier | *Ploiarium* (*B*) | 7691 |  | 12/04/2019 | 150 | HBV | AS |
| ******* | *B. horizontale* Bosser | *Kainochilus* (*A*) | 7765 |  | 08/02/2018 | 15 | M | AG, AS, DP |
| ******* | *B. humbertii* Schltr. | *Ploiarium* (*B*) | ORCH000463 |  | 29/06/2017 | 10 | HBV | AS |
| ******* | *B. jackyi* G.A.Fischer, Sieder & P.J. Cribb | *Ploiarium* (*B*) | 7051 |  | 01/03/2018 | 15 | HBV | AS |
| ******* | *B. jeanbosseri* Gamisch & Hermans | *Kainochilus* (*A*) | 7342 |  | 02/08/2018 | 30 | HBV | AS |
| ******* | *B. liparidioides* Schltr. | *Pachyclamys* (*B*) | ORCH000359 |  | 03/08/2017 | 15 | HBV | AS |
| ******* | *B. lucidum* Schltr. | *Ploiarium* (*B*) | S 2704 |  | 17/07/2017 | 15 | HBV | AS |
| ******* | *B. multiflorum* Ridl. | *Loxosepalum* (*B*) | 7378 |  | 19/01/2018 | 30 | M | AG, AS, DP |
| ******* | *B. multiligulatum* H.Perrier | *Kainochilus* (*A*) | 5612 |  | 15/01/2018 | 10 | M | AG, AS, DP |
| ******* | *B. namoronae* Bosser (1) | *Ploiarium* (*B*) | FS 6600 |  | 18/08/2017 | 20 | HBV | AS |
|  | *–* (2) |  | FS 6600 |  | 27/08/2019 | 150 | HBV | AS |
| ******* | *B. obtusatum* (Jum. & H.Perrier) Schltr. (1) | *Calamaria* (*A*) | FS 7137 |  | 18/04/2017 | 5 | HBV | AS |
|  | *–* (2) |  | FS 7137 |  | 18/04/2017 | 20 | HBV | AS |
| ******* | *B. oxycalyx* Schltr. | *Elasmotopus* (*B*) | 7619 |  | 03/02/2018 | 10 | M | AG, AS, DP |
| ******* | *B. paleiferum* Schltr. (1) | *Ploiarium* (*B*) | FS 5063 |  | 31/07/2018 | 20 | HBV | AS |
|  | *–* (2) |  | 5063 |  | 28/08/2019 | 150 | HBV | AS |
| ******* | *B. pantoblepharon* Schltr. | *Trichopus* (*B*) | 7526 |  | 31/01/2018 | 15 | M | AG, AS, DP |
| ******* | *B. pusillum* Thouars (1) | *Calamaria* (*A*) | S 6794 |  | 17/07/2017 | 15 | HBV | AS |
|  | *–* (2) |  | 7520 |  | 09/03/2018 | 20 | HBV | AS |
| ******* | *B. sarcorhachis* Schltr. | *Ploiarium* (*B*) | FS 1610 |  | 19/02/2019 | 150 | HBV | AS |
| ******* | *B. senghasii* G.A.Fischer & Sieder (1) | *Calamaria* (*A*) | FS 7210 |  | 29/06/2017 | 15 | HBV | AS |
|  | *–* (2) |  | ORCH110820 |  | 30/01/2019 | 150 | HBV | AS |
| ******* | *B. trifarium* Rolfe | *Calamaria* (*A*) | FS 6310 |  | 17/05/2017 | 30 | HBV | AS |
| ******* | *B. vakonae* Hermans | *Loxosepalum* (*B*) | 7664 |  | 05/02/2018 | 5 | M | AG, AS, DP |
| ******* | *B. sp. nov.* 1 | *Calamaria* (*A*) | FS 5383 |  | 05/03/2018 | 30 | HBS | SA |
|  | *B. sp.* | *Calamaria* (*A*) | 20792 |  | 29/06/2017 | 15 | HBV | AS |
|  | *B. sp.* 8013-1 | *Calamaria* (*A*) | 8013-1 |  | 16/08/2019 | 150 | HBV | AS |
|  | *B. sp.* 1525 | *Elasmotopus* (*B*) | 1525 |  | 27/08/2019 | 150 | HBV | AS |
|  | *B. sp.* FS 4397 | *Ploiarium* (*B*) | FS 4397 |  | 18/05/2017 | 15 | HBV | AS |
| ******* | *B. sp.* FS 4420 (1) | *Ploiarium* (*B*) | FS 4420 |  | 29/06/2017 | 15 | HBV | AS |
|  | *–* (2) |  | FS 4420 |  | 26/06/2019 | 150 | HBV | AS |
|  | *B. sp.* FS 7491 | *Ploiarium* (*B*) | FS 7491 |  | 10/05/2018 | 30 | HBV | AS |
|  | *B. sp.* 6755 | *Ploiarium* (*B*) | 6755 |  | 29/01/2019 | 150 | HBV | AS |
|  | *B. sp.* 7674 | *Ploiarium* (*B*) | 7674 |  | 11/03/2019 | 150 | HBV | AS |
|  | *B. sp.* OR 36_98 | *Ploiarium* (*B*) | OR 36_98 |  | Unknown | 30 | HBS | SA |
|  | *B. sp.* OR 83_2003 | *Ploiarium* (*B*) | OR 83_2003 |  | Unknown | 30 | HBS | SA |

^a^ Site codes: HBS, Botanical Garden of Salzburg University; HBV, Botanical Garden of Vienna University; M, Madagascar.

^b^ Collector abbreviations: AG, Alexander Gamisch; AS, Anton Sieder; DP, David Prehsler; SA, Silvia Artuso.

**Table S2** Evolutionary model fitting to each of the shared 59 floral volatile organic compounds (VOCs) of 32 Malagasy Bulbophyllum, using geiger. Each model (i.e. BM, Brownian motion; EB, early burst; OU, single-optimum Ornstein–Uhlenbeck) was evaluated over the time-calibrated maximum clade credibility (MCC) tree of Malagasy Bulbophyllum (Gamisch et al., 2021) pruned to these 32 species for which shared VOC data could be obtained in the present study (see also Fig. S1).

| VOC | AICc | | |  | ΔAICc | | | | Best model(s) | Best model parameter(s) |
| --- | --- | --- | --- | --- | --- | --- | --- | --- | --- | --- |
|  | BM | OU | EB |  | | BM | OU | EB |  |  |
| 3-Methylbutanoic acid* | 180.73 | 159.26 | 160.12 |  | | 21.48 | 0.00 | 0.86 | OU | *α* = 1.09 |
| 2-Methylbutanoic acid* | -38.89 | -62.85 | -61.23 |  | | 23.95 | 0.00 | 1.61 | OU | *α* = 1.49 |
| 1-Octen-3-ol* | 208.69 | 210.13 | 210.13 |  | | 0.00 | 1.44 | 1.44 | BM | *σ*^2^ = 7.36 |
| 6-Methyl-5-hepten-2-one* | 241.49 | 242.61 | 242.61 |  | | 0.00 | 1.12 | 1.12 | BM | *σ*^2^ = 20.52 |
| (*E*)-β-Ocimene* | 117.86 | 116.53 | 116.53 |  | | 1.32 | 0.00 | 0.00 | OU/EB | *α* = 0.21/*r* = 0.43 |
| Undecane* | 329.57 | 298.81 | 302.47 |  | | 30.76 | 0.00 | 3.66 | OU | *α* = 1.67 |
| Linalool* | 321.20 | 280.04 | 289.70 |  | | 41.16 | 0.00 | 9.65 | OU | *α* = 10.0 |
| 2-Phenylethanol* | 192.08 | 167.04 | 169.77 |  | | 25.04 | 0.00 | 2.73 | OU | *α* = 3.00 |
| Methyl octanoate* | -35.56 | -34.32 | -34.32 |  | | 0.00 | 1.24 | 1.24 | BM | *σ*^2^ = 0.003 |
| (*E*,*E*)-2.6-Dimethyl-1,3,5,7-octatetraene | 271.73 | 235.20 | 241.15 |  | | 36.53 | 0.00 | 5.95 | OU | *α* = 4.74 |
| Octanoic acid* | 289.44 | 282.54 | 282.57 |  | | 6.90 | 0.00 | 0.03 | OU | *α* = 0.60 |
| Methyl salicylate* | 184.26 | 154.79 | 158.26 |  | | 29.48 | 0.00 | 3.47 | OU | *α* = 2.66 |
| 2-Aminobenzaldehyde* | 69.21 | 53.26 | 53.26 |  | | 15.95 | 0.00 | 0.00 | OU/EB | *α* = 0.45/*r* = 0.89 |
| unk_1245 | 171.60 | 149.36 | 150.56 |  | | 22.24 | 0.00 | 1.20 | OU | *α* = 1.11 |
| Geraniol* | 274.88 | 273.92 | 273.92 |  | | 0.96 | 0.00 | 0.00 | OU/EB | *α* = 0.19/ *r* = 0.38 |
| 3,5-Dimethoxytoluene* | 149.98 | 122.27 | 124.88 |  | | 27.71 | 0.00 | 2.61 | OU | *α* = 3.48 |
| unk_1281 | 201.37 | 179.17 | 180.35 |  | | 22.20 | 0.00 | 1.18 | OU | *α* = 1.11 |
| Tridecene isomer 1 | -113.11 | -129.86 | -129.49 |  | | 16.75 | 0.00 | 0.38 | OU | *α* = 1.28 |
| Tridecadiene isomer | -39.11 | -56.51 | -56.02 |  | | 17.41 | 0.00 | 0.49 | OU | *α* = 1.38 |
| Tridecene isomer 2 | 160.49 | 143.58 | 143.64 |  | | 16.92 | 0.00 | 0.06 | OU | *α* = 1.66 |
| Tridecane* | 322.96 | 307.47 | 309.29 |  | | 15.48 | 0.00 | 1.82 | OU | *α* = 6.10 |
| Indole* | 282.28 | 256.92 | 257.90 |  | | 25.36 | 0.00 | 0.98 | OU | *α* = 0.76 |
| Methyl 2-hydroxy-4/5/or 6-methylbenzoate | 215.53 | 197.94 | 198.77 |  | | 17.60 | 0.00 | 0.83 | OU | *α* = 1.08 |
| Methyl anthranilate* | 93.00 | 77.92 | 77.92 |  | | 15.08 | 0.00 | 0.00 | OU/EB | *α* = 0.43/ *r* = 0.86 |
| Decanoic acid* | 76.10 | 42.79 | 48.01 |  | | 33.31 | 0.00 | 5.22 | OU | *α* = 4.73 |
| unk_1376 | 201.55 | 186.28 | 186.73 |  | | 15.27 | 0.00 | 0.45 | OU | *α* = 0.87 |
| Geranyl acetate* | 86.98 | 56.51 | 60.59 |  | | 30.47 | 0.00 | 4.08 | OU | *α* = 10.0 |
| Tetradecene isomer 1 | -66.44 | -64.00 | -74.83 |  | | 8.38 | 10.83 | 0.00 | EB | *r* = -0.27 |
| Tetradecene isomer 2 | -24.33 | -43.60 | -42.07 |  | | 19.27 | 0.00 | 1.53 | OU | *α* = 1.75 |
| α-Copaene* | -3.94 | -40.04 | -34.05 |  | | 36.10 | 0.00 | 5.99 | OU | *α* = 4.46 |
| N-Formyl-2-aminobenzaldehyde | 211.22 | 188.25 | 188.69 |  | | 22.97 | 0.00 | 0.44 | OU | *α* = 0.65 |
| unk_1430 | -33.43 | -69.60 | -63.78 |  | | 36.16 | 0.00 | 5.81 | OU | *α* = 5.21 |
| β-Caryophyllene | 252.30 | 241.39 | 241.39 |  | | 10.92 | 0.00 | 0.00 | OU/EB | *α* = 0.50/ *r* = 0.99 |
| unk_1450 | 219.29 | 213.32 | 213.32 |  | | 5.96 | 0.00 | 0.00 | OU/EB | *α* = 0.28/ *r* = 0.56 |
| (*E*)-β-Farnesene* | 184.48 | 153.40 | 157.99 |  | | 31.08 | 0.00 | 4.59 | OU | *α* = 4.54 |
| Pentadecadiene isomer | 273.77 | 246.99 | 249.50 |  | | 26.78 | 0.00 | 2.51 | OU | *α* = 2.23 |
| α-Caryophyllene* | 183.85 | 156.98 | 160.50 |  | | 26.86 | 0.00 | 3.52 | OU | *α* = 4.17 |
| Pentadecene isomer 1 | 214.05 | 216.50 | 64.62 |  | | 149.43 | 151.88 | 0.00 | EB | *r* = -1.0 |
| Pentadecene isomer 2 | -28.58 | -26.14 | -65.04 |  | | 36.46 | 38.90 | 0.00 | EB | *r* = -0.48 |
| unk_1496 | 93.03 | 63.76 | 67.11 |  | | 29.27 | 0.00 | 3.35 | OU | *α* = 2.41 |
| Pentadecane* | 195.34 | 186.07 | 187.31 |  | | 9.27 | 0.00 | 1.24 | OU | *α* = 3.01 |
| Germacrene D* | 264.35 | 266.80 | 253.19 |  | | 11.16 | 13.61 | 0.00 | EB | *r* = -1.0 |
| unk_1507 | 249.13 | 224.11 | 224.89 |  | | 25.02 | 0.00 | 0.78 | OU | *α* = 0.73 |
| α-Selinene* | 74.65 | 77.09 | 75.74 |  | | 0.00 | 2.44 | 1.10 | BM | *σ*^2^ = 0.11 |
| (*E,E*)-α-Farnesene | 223.90 | 205.55 | 206.85 |  | | 18.35 | 0.00 | 1.29 | OU | *α* = 1.91 |
| β-Selinene* | -10.22 | -7.77 | -33.11 |  | | 22.90 | 25.34 | 0.00 | EB | *r* = -0.33 |
| β-Bisabolene | 56.06 | 31.24 | 32.07 |  | | 24.82 | 0.00 | 0.84 | OU | *α* = 0.73 |
| unk_1522 | 42.10 | 21.40 | 23.68 |  | | 20.71 | 0.00 | 2.29 | OU | *α* = 2.11 |
| unk_1528 | 82.72 | 46.30 | 52.32 |  | | 36.42 | 0.00 | 6.01 | OU | *α* = 5.07 |
| unk_1545 | 187.91 | 164.61 | 165.29 |  | | 23.29 | 0.00 | 0.67 | OU | *α* = 0.72 |
| unk_1570 | 259.72 | 238.93 | 240.25 |  | | 20.79 | 0.00 | 1.32 | OU | *α* = 1.33 |
| (*E*)-Nerolidol* | 77.88 | 58.39 | 58.56 |  | | 19.49 | 0.00 | 0.17 | OU | *α* = 0.61 |
| unk_1577 | 171.07 | 148.80 | 150.27 |  | | 22.27 | 0.00 | 1.47 | OU | *α* = 1.29 |
| Caryophyllene oxide* | 167.24 | 150.54 | 151.14 |  | | 16.71 | 0.00 | 0.61 | OU | *α* = 0.99 |
| Humulene epoxide isomer | 45.51 | 26.41 | 27.83 |  | | 19.10 | 0.00 | 1.42 | OU | *α* = 3.39 |
| Heptadecadiene isomer | -103.80 | -101.35 | -105.72 |  | | 1.92 | 4.36 | 0.00 | EB | *r* = -0.18 |
| Heptadecene isomer | 114.02 | 116.47 | 13.36 |  | | 100.66 | 103.10 | 0.00 | EB | *r* = -0.84 |
| unk_1707 | 138.93 | 116.06 | 117.58 |  | | 22.88 | 0.00 | 1.52 | OU | *α* = 1.28 |
| Hexahydrofarnesylacetone | 160.79 | 152.30 | 152.30 |  | | 8.48 | 0.00 | 0.00 | OU/EB | *α* = 0.37/ *r* = 0.75 |

AICc, Akaike Information Criterion, corrected for small sample data; ΔAICc, difference in AICc value between the best model and the model being compared. Model parameters: *α*, rate of adaptation parameter for OU; *r*, EB parameter of exponential rate decrease; *σ*^2^⁠, diffusion parameter of the univariate BM model. * indicates compounds indentified based on synthetic compounds.

**Methods S1** Identification of a novel floral scent compound, N,N-dimethylleucine O-methyl ester, using Nuclear Magnetic Resonance (NMR) spectroscopy.

A previously unknown floral scent compound [mass-to-charge (m/z) values (relative intensity, %): 42(6), 58(12), 72(34), 114(100), 115(8), 116(12)] dominating the field-collected scent of a *Bulbophyllum cardiobulbum* (clade *D*) specimen (voucher 7439; see Table S1) was analysed by Nuclear Magnetic Resonance (NMR) spectroscopy. To this aim, the floral scent of *B. cardiobulbum* from the same specimen plant cultivated at the Botanical Garden of Vienna University (HBV), was sampled again as described in the main text, but with a larger adsorbent tube (glass capillary, length = 8 cm, inner diameter = 2.5 mm) filled with 15 mg Tenax-TA (mesh 60-80) and 15 mg Carbotrap B (mesh 20-40). Scent was collected in this tube eight times for four hours each from a single flower. The volatiles trapped in the adsorbent tube were eluted with 750 μl of deuterated aceton-d6 (Armar Europe, Cunnersdorf, Germany) and measured in a standard 5 mm NMR tube, TA quality (Armar), on a 600 MHz Avance III HD spectrometer (Bruker Biospin, Rheinstetten, Germany) equipped with a QXI (^1^H/^13^C/^15^N/^31^P) probe at 298 K. Chemical shift assignment (Table S3) was achieved using standard one-dimensional ^1^H and two-dimensional ^1^H-^1^H TOCSY, ^1^H-^1^H COSY, ^1^H-^13^C HSQC and ^1^H-^13^C HMBC spectra (Fig. S3). Spectra were processed by topspin v.3.2 (Bruker Biospin) and referenced to tetramethylsilane (TMS). Spectra were analyzed using the software sparky v.3.114 (Goodard & Keller, 2007).

**Table S3** Observed chemical shifts of N,N-dimethylleucine O-methyl ester observed in acetone-d6 at 298 K, referenced to tetramethylsilane (TMS) in comparison with values of similar or identical molecules from the literature. Atom numbering is given in Figure S3.

| Chemical shift assignments | | | | | | | | | | | | | | | | Reference |
| --- | --- | --- | --- | --- | --- | --- | --- | --- | --- | --- | --- | --- | --- | --- | --- | --- |
| H2 | H3 | H3' | H4 | H5 | H5' | H6/H6' | H7 | C1 | C2 | C3 | C4 | C5 | C5' | C6 | C7 |  |
| 3.24 | 1.53 | 1.48 | 1.64 | 0.92 | 0.88 | 2.28 | 3.64 | 172.9 | 65.4 | 39.1 | 25.3 | 23.1 | 22.2 | 41.2 | n.d. | This study |
| 3.23 | 1.60 | 1.60 | 1.50 | 0.92 | 0.90 | 2.32 | 3.70 | n.r. | n.r. | n.r. | n.r. | n.r. | n.r. | n.r. | n.r. | Kaiser (1986) ^a^ |
| 3.15 | 1.52* | 1.41* | 1.71* | 0.88 | 0.88 | 2.48 | – | n.r. | n.r. | n.r. | n.r. | n.r. | n.r. | n.r. | – | Tajbakhsh *et al.* (2011) ^b^ |
| 4.65 | 1.55 | 1.08 | 0.94 | 0.63 | 0.55 | 2.88 | – | 170.1 | 52.6 | 38.2 | 24.7 | 23.0 | 22.9 | 30.9 | – | Cruz *et al*. (2006) ^c^ |
| 4.34 | 1.62 | 1.62 | 1.59 | 0.92 | 0.87 | – | – | 174.9 | 52.4 | 39.7 | 24.4 | 22.2 | 20.6 | – | – | Wishart *et al.* (1995) ^d^ |

n.r., not reported values; n.d., not determined values; –, this atom does not exist in the entity.

^a^ Data of N,N-dimethylleucine O-methyl ester; the solvent was not mentioned in Kaiser (1986).

^b^ Data of N,N-dimethylleucine measured (lacking the ester) in DMSO-d6; no assignments were given in Tajbakhsh *et al.* (2011); here we assigned the reported values to the best fitting observed values; values with an asterisk were given as a range, e.g. 1.74–1.67 ppm, and for simplicity we report in the table the average value of both limits.

^c^ Data of N,N-dimethylleucine as the N-terminal residue of a cyclodepsipeptide measured in CDCl_3_.

^d^ Random coil chemical shifts of leucine as part of a peptide (lacking N- and O-methylation) measured at 25 °C, in D_2_O in the presence of 1 M urea-d4 and 50 mM phosphate pH 5.1; ^13^C values originally referenced to disuccinimidyl suberate (DSS) were recalibrated to TMS by subtracting 2.66 ppm.

**Fig. S3** Identification of N,N-dimethylleucine O-methyl ester by NMR spectroscopy. (a) Chemical structure and used nomenclature of N,N-dimethylleucine O-methyl ester. (b) The most important observed correlations in 2D ^1^H-^1^H COSY and 2D ^1^H-^13^C HMBC spectra, as illustrated by arrows on the chemical structure. (c) Selected regions of a 2D ^1^H-^13^C HMBC spectrum showing key correlations.


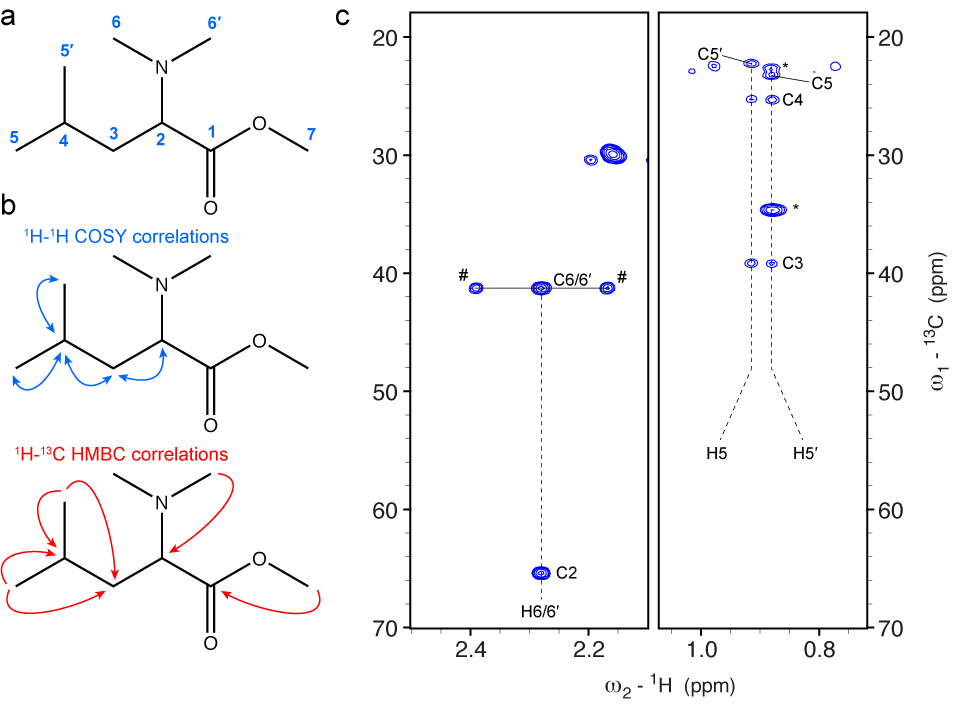


**References**

**Cruz LJ, Insua MM, Baz JP, Trujillo M, Rodriguez-Mias RA, Oliveira E, Giralt E, Albericio F, Cañedo LM. 2006.** IB-01212, a new cytotoxic cyclodepsipeptide isolated from the marine fungus *Clonostachys* sp. ESNA-A009. *The Journal of Organic Chemistry* **71**: 3335–3338.

**Gamisch A, Winter K, Fischer GA, Comes HP. 2021.** Evolution of Crassulacean Acid Metabolism (CAM) as an escape from ecological niche conservatism in Malagasy *Bulbophyllum* (Orchidaceae). *New Phytologist* **231**: 1236–1248.

**Goddard TD, Kneller DG. 2007.** SPARKY 3 (v3.114, Windows). San Francisco, CA, USA: University of California. [WWW document] URL <https://www.cgl.ucsf.edu/home/sparky/> [last accessed 31 March 2025].

**Kaiser R. 1986.** New natural products of structural and olfactory interest identified in fig leaf absolute (*Ficus carica* L.). In:  Brunke E-J, ed. *Progress in essential oil research*. Berlin, Germany: Walter de Gruyter, Berlin, 227–239.

**Tajbakhsh M, Hosseinzadeh R, Alinezhad H, Ghahari S, Heydari A, Khaksar S. 2011**. Catalyst-free one-pot alkylation of primary and secondary amines and N,N-dimethylation of amino acids using sodium borohydride in 2,2,2-trifluoroethanol. *Synthesis* **3**: 0490–0496.

**Wishart DS, Bigam CG, Holm A, Hodges RS, Sykes BD. 1995.** ^1^H, ^13^C and ^15^N random coil NMR chemical shifts of the common amino acids. I. Investigations of nearest-neighbor effects. *Journal of Biomolecular NMR* **5**: 67–81.
